# A fire risk pre-warning framework for high-rise buildings based on unascertained method

**DOI:** 10.1007/s11356-024-35396-y

**Published:** 2024-10-25

**Authors:** Li-Ning Zhang, Xiang-Ming Wang, Jing An, Hong Xian Li, Jiao-Qian Guo, Guo-bo Han, Peng-Fei Gou

**Affiliations:** 1 School of Architecture Engineering, North China Institute of Science and Technology, Beijing, 101601 China; 2 School of Project Management, The University of Sydney, Sydney, NSW 2006 Australia; 3 School of Electronic Information Engineering, North China Institute of Science and Technology, Beijing, 101601 China; 4 School of Architecture and Built Environment, Deakin University, Geelong, 3220 Australia; 5 School of Mechanical and Electrical Engineering, Beijing University of Technology, Beijing, 100081 China

**Keywords:** High-rise buildings, Pre-warning tree for fire prevention risk, Unascertained pre-warning modeling, Case study

## Abstract

The growing global interest in preventing and controlling fires in high-rise buildings reflects the increasing significance of this issue today. This research aims to establish an early warning framework for fire risk in high-rise buildings. Firstly, considering the importance of a scientific indicator system for the application of the model, this study combines the event analysis method with the building design fire code to identify 11 key risk factors that have a far-reaching impact on the prevention of fires in high-rise buildings. Based on identifying the risk factors, a high-rise building fire risk warning tree is also established, which scientifically solves the problem of the indicator system of the warning object. Subsequently, in response to the various complex issues arising from the uncertainty of fire occurrence in high-rise buildings, this study adopts the unascertained method to model the fire risk of high-rise buildings for early warning. In addition, the developed methodology was empirically validated through case studies and analyses of empirical data on fire risks in nine representative high-rise buildings. The results of the unascertained method were also compared with the results of the K-means method, from which it was concluded that the unascertained method can predict building fires more accurately. The research results provide a reliable decision support system for fire disaster prevention and control in high-rise buildings.

## Introduction

Addressing and managing fires in high-rise buildings remain a persistent global challenge (Tan and Moinuddin 2019). Owing to its architectural characteristics, the consequences of a fire in a high-rise building could be extremely serious in the event that it occurs. For instance, a fire in a 24-story apartment building in London in 2017 resulted in 80 deaths. Similarly, a fire in a high-rise building in Taiwan in 2021 killed 46 people and injured 41, while a fire in a high-rise apartment building in New York in 2022 resulted in 19 fatalities. On February 23, 2024, a fire broke out in a 34-story high-rise residential building in Nanjing, causing 15 deaths and 44 injuries (France-Presse 2024). The maximum working height of the fire ladder truck is usually limited to 100 m, a constraint that makes it difficult for the external fire department to effectively rescue the trapped occupants in the event of a fire in a high-rise building (Aleksandrov et al. 2019). In light of this, experts have observed that more emphasis should be placed on self-rescue measures for buildings higher than 50 m. The research shows that 46.2% of existing high-rise buildings in China need to be equipped with automatic fire protection facilities (Rahardjo and Prihanton 2020).

For these reasons, fire prevention in high-rise buildings is of critical importance. Fire prevention capability in high-rise civil buildings refers to the ability to prevent fire or to prevent the fire from spreading once it occurs in high-rise buildings. The study establishes a fire prevention risk pre-warning model for high-rise buildings to reduce the incidence and extent of fire disasters and associated losses.

There are several representative methods of fire prevention risk pre-warning for high-rise buildings. First, analytic hierarchy process (AHP) modeling to assess the fire risk of high-rise buildings (Li et al. 2018). Despite the strong practical value of this method, the method can effectively integrate multiple risk factors and provide a structure for building fire risk analysis, but the model relies on the expert’s experience to make the judgment, which may affect the validity of the analysis results. In addition, building information modeling for building fire prevention and disaster relief is also an effective approach (Lotfi et al. 2021). Nevertheless, this approach relies heavily on accurate and comprehensive data, and if data from different sources are inconsistent or incomplete, this poses a serious challenge to the accuracy of the results of the analysis. In, addition, a number of modeling approaches have also been successful in providing viable solutions for risk pre-warning of high-rise buildings, such as multi-story area modeling for building fire risk assessment (Himoto and Suzuki 2021), structural entropy weights modeling for fire risk assessment in large commercial buildings (Liu et al. 2017), and fuzzy modeling based on thermal imagery for potential classification and identification of fire outbreaks (Sousa et al. 2019). While these methods have provided assistance, the drawbacks are similarly obvious. These models are computationally intensive and are not suitable for use in complex high-rise buildings. Finally, there are also representative methods for pre-warning of the risk of fire prevention in high-rise buildings, such as the use of the ensemble Kalman filter method for real-time building fire forecasting (Ji et al. 2018), and neural network modeling (Wang et al. 2021). These methods provide real-time, high-precision fire risk prediction, but the high computational complexity of the model and its computational demands have an impact on the applicability of the method.

In addition, recent research on fire prevention and pre-warning has emphasized the critical role of warning object indicators in determining the effectiveness of early warning systems, especially in the context of fire prevention in high-rise buildings (Koutsomarkos et al. 2021). However, there are notable disparities in evaluation criteria; some are overly complicated, while others could be more complex, decreasing the consistency in high-rise building fire prevention risk pre-warning. These indicators are essential elements that underpin the accuracy and reliability of early warning mechanisms and are crucial for protecting occupants and mitigating potential fire risks in high-rise buildings.

Therefore, the establishment of an objective space of forecast object characteristics (i.e., the set of evaluation indicators) through the scientific extraction of forecast object characteristics (indicators) plays a decisive role in the accuracy of fire risk forecasting for high-rise buildings. A study by Li et al. (2018) argued that fire safety hardware facilities, building fire safety evacuation capability, building fire performance, and safety status of fire safety in construction units are all vital reference indices for fire prevention warnings in high-rise buildings. In addition, the complexities of the internal of a building, such as fire load density, are essential points of reference for the development of fire warning calculation models (Omar et al. 2023). These studies have analyzed the reference factors for the modeling of fires in high-rise buildings. However, the research methods employed in these studies tend to focus on specific cases or regions, thus limiting the generalizability and accuracy of the given model. For example, the study conducted by Omar et al. (2023) relies primarily on literature reviews and government data and does not consider detailed building-specific cases. This limitation directly affects the efficacy of the early warning system. In light of these concerns, the present study endeavors to construct a comprehensive fire prevention risk pre-warning framework for high-rise structures encompassing fire prevention, control, and emergency management by integrating event analysis techniques with unascertained modeling.

This study provides contributions to both theoretical research and the industry’s development. Firstly, in terms of theoretical research, this study collected and analyzed fire risk data from nine high-rise buildings, which provided the database for the models in this study and will be used as open information to help other researchers optimize the fire risk assessment models. In addition, the risk warning model and the analysis process developed in this study will serve as an empirical reference to help other researchers conduct fire risk assessments for different types of high-rise buildings. In terms of industry development, the uncertainty model used in this study improves the accuracy and reliability of predictions, which guides managers of high-rise buildings to take the necessary precautions before a fire occurs. In addition, this study aims to provide a scientific and systematic approach to fire risk assessment in high-rise buildings, and the results of this study will contribute to the standardization of fire risk management, which will enhance the overall prevention and control level of the industry.

In the next section, this study will specify the research methodology and introduce 11 main factors contributing to fires in high-rise buildings. After that, this study will introduce the uncertainty pre-warning modeling in detail and elaborate on the use of this model and the calculation process in the “Methodology” section. In the “Case study” section, nine typical high-rise buildings are selected and simulated using the uncertainty pre-warning modeling. By comparing the calculation results of the modeling with those of the K-means clustering approach, this study verifies the effectiveness and accuracy of the pre-warning modeling. Finally, the research concludes with a summary of the results in the “Discussion and conclusion” section.

## Methodology

**Fig. 1:**
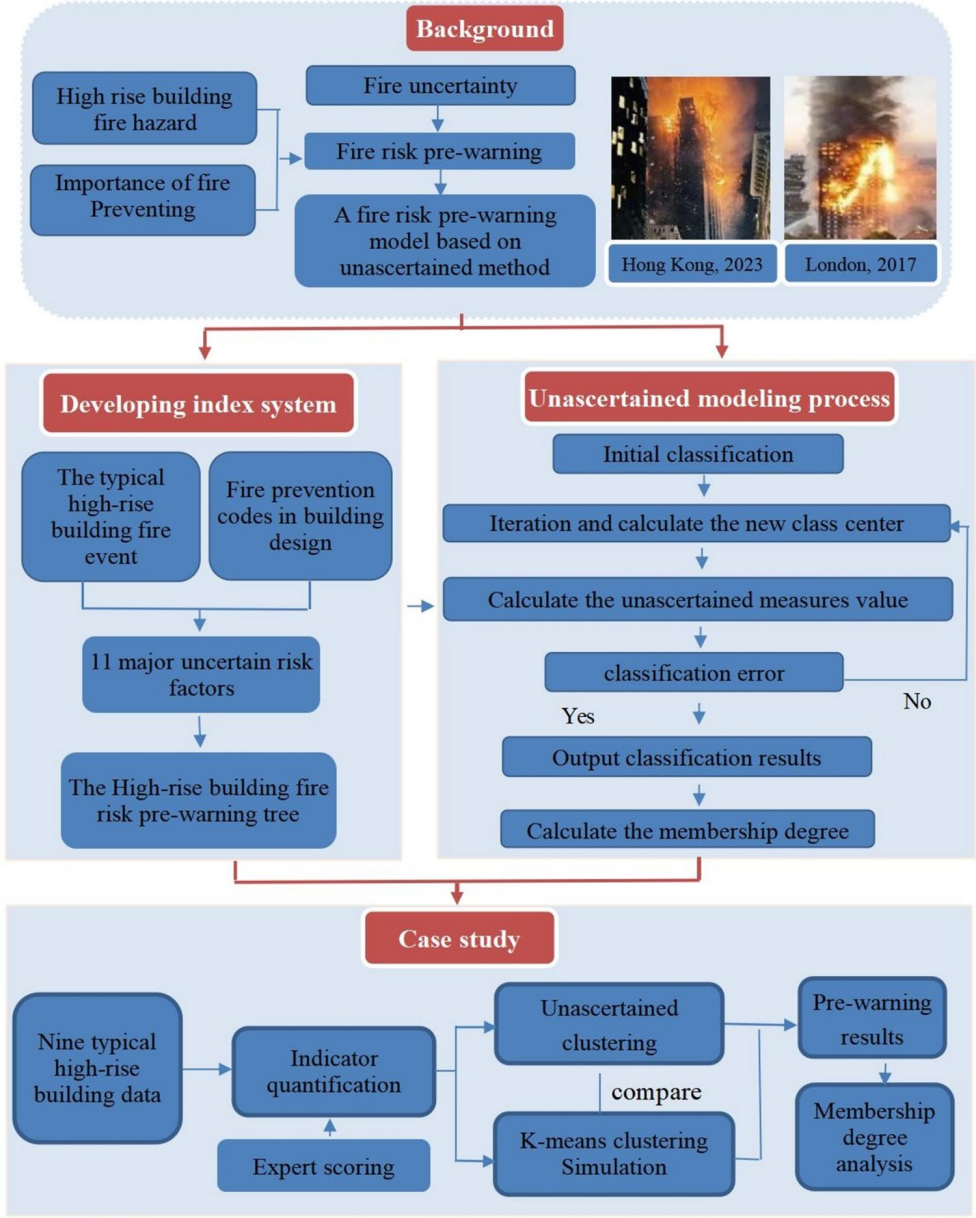
Overview of research methodology

This study uses a mixed method of literature review, uncertainty modeling techniques, and case study to establish an early-warning framework of fire risk in high-rise buildings. A mixed research approach combines the strengths of quantitative and qualitative research to provide a comprehensive and in-depth analysis, thereby increasing the credibility and reliability of the findings (Heyvaert et al. 2013). By analyzing typical cases of high-rise building fires and fire codes, this study identifies eleven major fire risk factors. This research system ensures the validity, systematicity, and scientificity of the factors. In addition, the factors analyzed in this study cover a wide range of risk factors such as fire resistance, fire load, and building layout, which ensures the practicality and comprehensiveness of the factors. Besides, this study establishes a fire risk pre-warning tree for high-rise buildings. Based on this pre-warning tree, this study uses uncertainty modeling techniques to model the fire risk and provide early warning of the fire risk for high-rise buildings. This fire early warning framework addresses the various complexities of uncertainty in the occurrence of fires in high-rise buildings. Finally, this research conducts a case study using the data from nine representative high-rise buildings. By comparing the outcomes of the early warning system with those obtained from the K-means clustering approach, this research confirms the efficacy of the fire risk early warning system. The research methodology is shown in Fig. 1.

### A fire risk pre-warning tree for high-rise buildings

This study identifies the main fire risk factors based on typical events and building fire codes to construct a fire risk pre-warning tree for high-rise buildings, where real building fire cases support each indicator. Based on the extraction of the main factors, a fire risk warning tree is then established for high-rise buildings. The construction process of the fire risk pre-warning tree based on typical events and building fire code analysis is conducted as follows.

#### Main factors affecting the general layout of high-rise buildings

Building layout has a direct impact on fire prevention and building safety, and this impact is particularly evident in high-rise buildings. The way a building is designed and organized is closely related to the ease of evacuation in the event of a hazard, the degree of fire spread, and the effectiveness of the fire protection system (Nimlyat et al. 2017). For example, the layout has a significant impact on critical elements such as the design of smoke evacuation systems, the placement of automatic alarms, and the configuration of stairways, all of which have a direct impact on the ability to warn of a fire at an early stage and the efficiency of the evacuation procedure in the event of a fire (Song et al. 2022).

Vertical evacuation in high-rise buildings can be complex and challenging, making the layout of high-rise buildings critical to building fire protection. Optimized evacuation routes and well-located escape stairwell layouts are important components of high-rise building design and are essential for the safety of occupants in the case of fire (Ronchi and Nilsson 2013). In addition, the surroundings of a high-rise building and fire distances are essential factors in limiting the impact of a fire. An open perimeter and shorter distances between the building and the fire station improve fire control efficiency and minimize the fire’s potential adverse effect on the evacuation of occupants (Nimlyat et al. 2017).

Several case studies have explored the impact of building layout on fire events, revealing the importance of building layout planning and design. For example, the Sun Department Store fire in Kumamoto Prefecture, Japan (1973), the Nairobi City Office Building fire in Nairobi, Kenya (1979), and the Swan Hotel fire in Harbin, China (1985), have been regarded as successful stories. In each case, the rational layout positively affected building fire prevention, facilitating easy access for fire engines and rescue personnel and allowing rapid intervention at the main fire points, thereby minimizing fire damage.

In contrast, the Busan high-rise hotel fire (1984) is an example of the consequences of poor building layout, where the poorly planned layout hampered firefighting efforts, leading to significant losses and challenging rescue operations.

The main factors affecting the general layout of high-rise buildings are as follows:

1. Fire separation distance: Representative events underscoring fire separation distance considerations include the Andraus Building fire in São Paulo, Brazil (1972). In this case, the local fire brigade arrived on the scene within 26 min, but by that time, under the influence of wind, the building’s front was engulfed in flames up to 40 m wide and 100 m high. The extended fire tongues and intense heat radiation reached two residential apartment buildings 30 m away, causing heavy losses. As this example demonstrates, a safe distance needs to be maintained between high-rise buildings and other buildings to prevent the fire from spreading through heat radiation or other means. Table 1 outlines the fire protection spacing provisions that have historically been in place for high-rise buildings (China Ministry of Public Security 2018). However, the actual fire protection spacing of new high-rise buildings is often different from standard requirements. For example, the building fire protection distance is 40 to 50 m in Beijing, Shanghai, and other large Chinese cities. Therefore, based on the fire code, an extended table (Table 2) is developed for quantifying fire prevention space in this research.
2. The building surrounding environment: Representative events in this regard include the “8.12” dangerous goods warehouse explosion in Tianjin, China (2015), which resulted in 55 deaths and hundreds of injuries to the occupants of surrounding residential buildings. In 2013, the “11.22” gas pipeline explosion in Qingdao, China, extended hundreds of meters, resulting in 62 deaths. As these examples emphasize, high-rise buildings should be as far as possible from surrounding significant hazards, such as factories or warehouses that produce or store inflammable or explosive materials. At the same time, other fire risk factors and the presence of a large number of combustibles in the surrounding environment also significantly influence the occurrence of high-rise building fires. The criteria for quantifying the building surrounding conditions are developed based on the fire code in Table 3 in this research.

**Table 1:** Fire prevention spacing of high-rise buildings

Building category	Minimum spacing between high-rise buildings (classes 1 and 2)	Minimum spacing between high-rise buildings and other types of buildings		
		Classes 1 and 2	Class 3	Class 4
Fire prevention spacing (m)	13	9	11	14

**Table 2:** Fire spacing indicator scoring of high-rise building

Building category		Fire spacing with other high-rise buildings (m)	Fire spacing with other types of buildings (m)			Actual risk condition	Value range
			Classes 1 and 2	Class 3	Class 4		
High-rise building	Main body	≥ 13	≥ 9	≥ 11	≥ 14	Safe	80 ~ 100
	Annex	≥ 9	≥ 6	≥ 7	≥ 9		
	Main body	12 ~ 13	8 ~ 9	10 ~ 11	13 ~ 14	Normal	60 ~ 79
	Annex	8 ~ 9	5 ~ 6	6 ~ 7	8 ~ 9		
	Main body	10 ~ 12	6 ~ 8	8 ~ 10	12 ~ 13	Dangerous	45 ~ 59
	Annex	6 ~ 8	3 ~ 5	4 ~ 6	6 ~ 8		
	Main body	< 10	< 6	< 8	< 12	Very dangerous	0 ~ 44
	Annex	< 6	< 3	< 4	< 6		

The range values include the lower limit but not the upper limit (value range excluded)

**Table 3:** Building surrounding environment scoring

Building surrounding condition	Actual risk condition	Value range
No flammable or explosive substances	Safe	80 ~ 100
No fire risk source		
No flammable or explosive substances	Normal	60 ~ 79
There are individual fire risk sources		
There are individual flammable and explosive substances, or a small number of fire-risk sources	Dangerous	45 ~ 59
There are more flammable and explosive materials, or a large number of fire-risk sources	Very dangerous	0 ~ 44

#### Main factors affecting building fire-resistance of high-rise buildings

The fire resistance of high-rise buildings is affected by various factors, including structure types and building interior materials (Phan et al. 2010). These factors affect the overall fire resistance of a building and the ability to ensure that the building maintains its structural integrity during a fire. For example, fire-resistant materials that are resistant to high temperatures will effectively slow the spread of fire and provide valuable time for evacuation. The type of structure in a high-rise building plays an important role in limiting fire spread and ensuring timely fire suppression. The fire of the Guangzhou Nanfang Building is a typical event related to the fire resistance of building structures, which are constructed with reinforced concrete structures and reach up to 90 h in fire (iMedia 2024). This case shows the importance of fire-resistance in high-rise buildings. Table 4 is used for quantifying the structural fire resistance of high-rise buildings in this research.

**Table 4:** Structure fire-resistant grade scoring of high-rise buildings

Structure fire-resistant grade	Actual risk condition	Value range
Class 1	Safe	80 ~ 100
Classes 1 and 2	Normal	60 ~ 79
Class 2	Dangerous	45 ~ 59
Below class 2	Very dangerous	0 ~ 44

A representative fire example involving interior materials in high-rise buildings was the 1980 Las Vegas MGM Grand Hotel fire. The fire was initially caused by an electrical malfunction in a restaurant inside the building, which contributed to a small fire. However, the materials inside the building fueled the fire and eventually caused it to get out of control. An investigation into the fire found that the building contained an unusually high level of flammable materials, which was one of the major reasons why the fire got out of control so quickly (Hensler 2020). The interior materials of high-rise buildings are a significant fire hazard. If the interior decorative materials of a building are flammable, the fire can spread rapidly and be difficult to extinguish. For this reason, decorative materials for high-rise buildings, such as those for ceilings, floors, and walls, have to be Class A or Class B1 materials. Table 11 in the appendix is used for quantifying the fire prevention grade and decorative materials smokiness of high-rise buildings in this research.

#### Main factors affecting electrical fire prevention of high-rise buildings

Electrical fire prevention is also one of the important factors affecting fire prevention in high-rise buildings. Statistics show that about one-third of building fires can be attributed to electrical causes, with fires caused by electrical installations topping the list (Simonson et al. 2000). Given the complex structure and limited spatial characteristics of high-rise buildings, great attention should be given to ensuring the reliability and safety of electrical systems and equipment. For this reason, power supply cables in high-rise buildings tend to have higher fire ratings than ordinary buildings (Nimlyat et al. 2017). In addition, electrical equipment in high-rise buildings tends to generate a considerable amount of heat during operation, and overloading or short-circuiting could generate arcs and sparks that may ignite nearby flammable materials and cause fires. As such, the reliability and safety requirements for electrical equipment and power distribution in high-rise buildings exceed those for ordinary buildings (Hu 2016). Representative events for this aspect of fire prevention in high-rise buildings include the Beijing Wanguocheng T3 Building fire (2008) caused by a short circuit in the electrical wiring. This contributes to the ignition of the wall insulation layer.

The reliability of electrical equipment is one of the main causes of electrical fire performance in high-rise buildings. For example, the 1997 Chrysler Building fire in New York was caused by a transformer fire in the building, while the 1996 fire in the Trade Center building in Milan, Italy, was caused by an electrical fault during renovation. These incidents emphasize the importance of ensuring the reliability of electrical equipment in high-rise buildings. Another important consideration in this regard is that a chain reaction caused by overheating electrical equipment can quickly escalate into a full-scale fire, posing a significant threat to building occupants, property, and the surrounding environment. Ensuring the reliability and safety of electrical systems and equipment in high-rise buildings is critical in reducing the likelihood of such catastrophic events. Careful measures should be taken to mitigate the risks associated with electrical failures and to protect these structures from potential fire hazards. Table 12 in the appendix is used for scoring the reliability of electrical equipment of high-rise buildings in this research.

In addition to the reliability of electrical equipment, the safety of power distribution equipment also affects the electrical fire performance of high-rise buildings. Representative examples of fire events involving power distribution equipment include the Tianqiao Hotel fire in Wuhan (2011). The fire began when an air conditioning switch installed on a wall overheated, igniting the moisture-proof layer of the wall and the wood finishing. In another example, in 1997, 15 people died in the Jakarta Bank Building fire, and it was later found that the fire was caused by an air conditioning short circuit. As these examples underscore, in addition to ensuring the reliability of the electrical equipment itself, other safety precautions, such as regular maintenance checks and unobstructed emergency exits, are essential to consider. Such measures are essential when the electrical equipment is close to combustible materials. Table 13 in the appendix is used for quantifying the safety facilities of power distribution equipment of high-rise buildings in this research.

The root cause of most electrical fires in high-rise buildings is the occurrence of a short circuit or overloading of the wiring, leading to overheating and subsequent fire. The Guangzhou Jianye Building fire was one of the most catastrophic electrical fires in high-rise buildings, causing a direct economic loss of about 40 million yuan. The cause of the fire was a short circuit caused by an electrician’s unauthorized work. Similarly, in 2012, a fire was caused by a short circuit in the external wall of the Baiyun Hotel in Guangzhou. Dense and often hidden cables in modern high-rise buildings pose a challenge in promptly identifying and resolving potential problems. In such cases, fires are difficult to contain and can spread rapidly. Ensuring the reliability of the distribution circuits, such as fire resistance of wires and cables, is therefore critical to preventing electrical fires in high-rise buildings. Table 14 in the appendix is used for quantifying the fire resistance of wires and cables of high-rise buildings in this research.

#### Main factors affecting fire load of high-rise buildings

Fire load refers to the amount of combustible material in a building and the total heat generated by the combustible material in the building as it burns. Fire load plays a crucial role in determining the heat release rate during a fire event and, thus, the severity of the event (Dundar and Selamet 2023). The fire protection design of high-rise buildings need take into account various factors in this regard—such as the types of materials used in construction, furnishings, equipment, and other factors that may contribute to the fire load—in order to minimize the potential damage that could be caused in the event of a fire.

Dundar and Selamet (2022) investigated 50 high-rise buildings in Istanbul, Turkey, and found that the current internal fire loads in the buildings range from 310 650 MJ/m^2^ depending on the location of the room, which is higher than Eurocode. The results of the study indicate that the time for the fire to spread in a building will increase with the fire load by between 1 and 11 min. Table 15 in the appendix is used for quantifying the fire load density scoring of high-rise buildings in this research.

#### Main factors affecting fire compartmentation of high-rise buildings

The fires in the Joma and Andraus buildings in Brazil, which caused 179 deaths and 300 injuries, underscore the importance of fire compartmentation, as these fires were due to inadequate fire compartmentation in the building design. Numerous studies have shown that a well-designed fire zoning system plays a crucial role in effectively keeping fires within a defined area of a high-rise building, creating conditions conducive to safe evacuation and fire suppression (Pershakov et al. 2016). For example, although the 300-m-high John Hancock Tower in Chicago has had at least 20 fires to date, it is noteworthy that none of these fires spread beyond the area of origin, mainly attributable to a proper fire compartmentation system. Similarly, while several fires have occurred at the Dubai Torch Tower in recent years, these were contained without loss of life due to effective fire separation measures. The strategic implementation of fire compartmentation ensures that any potential fires are confined to specific compartments or floors, stopping them from spreading quickly, providing enough time for occupants to evacuate safely and providing more time for the fire department to respond to the fire.

Table 5 presents the maximum permissible floor area for fire compartmentation in high-rise buildings in China, and these values are influenced by the presence or absence of automatic fire extinguishing systems. The table is intended to guide the size of the fire zoning of high-rise buildings to reduce the likelihood of large-scale fires from a design point of view. Therefore, based on the fire code, an extended table (Table 16) is developed for quantifying the horizontal and vertical fire division of high-rise buildings in this research.

**Table 5:** Maximum allowable building area of fire compartmentation in high-rise buildings

Buildings	Maximum allowable building area of fire compartmentation (m^2^)	
	No automatic fire extinguishing system	Automatic fire extinguishing system
High-rise buildings	1500	3000
Underground buildings (rooms)	500	1000

#### Main factors affecting smoke prevention and exhaust ability of high-rise buildings

Research shows that more than 60% of the deaths in fire accidents are caused by toxic smoke suffocation (Alianto et al. 2022). Therefore, effective smoke prevention and exhaust design are important measures for reducing casualties in high-rise building fires. Representative events in this regard include the Dongdu Shopping Mall fire in Luoyang, China (2000), in which 309 people were killed by smoke in the four-story entertainment center, even though the fire broke out on the second floor underground and did not spread to floors above the ground. Similarly, 67 of the 84 people who died in the MGM Grand Hotel fire suffocated to death. These examples demonstrate that smoke control and exhaust systems play an important role in the fire safety of high-rise buildings. Table 17 in the appendix is used for quantifying the intact condition of smoke prevention and exhaust facilities of high-rise buildings in this research.

According to the literature (Chen et al. 2018; Baalisampang et al. 2021), the smoke prevention and exhaust ability of high-rise buildings can be comprehensively evaluated from two perspectives: the intact condition of smoke prevention and exhaust facilities and the rational design of smoke prevention and exhaust systems. Table 18 in the appendix is used for quantifying the design rationality of smoke prevention and exhaust system of high-rise buildings in this research.

**Fig. 2:**
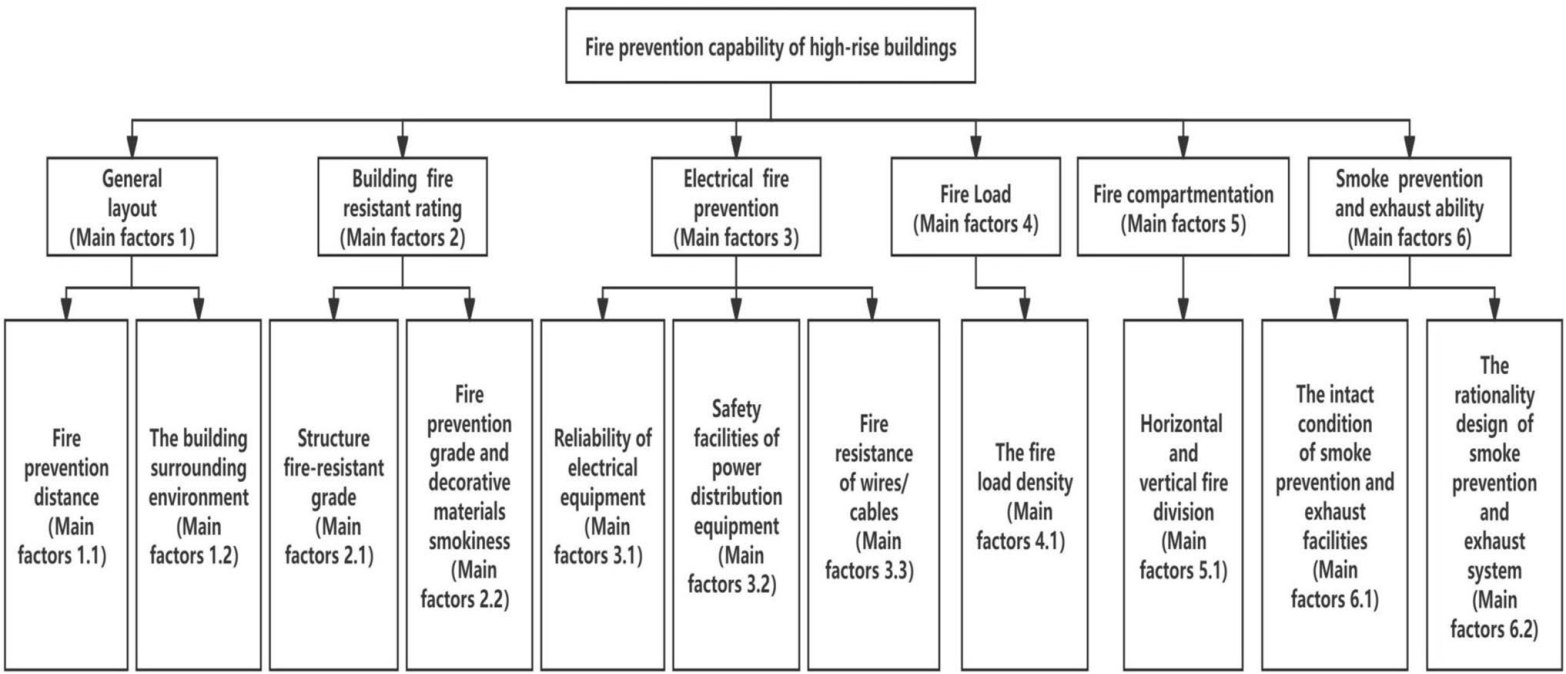
Fire prevention risk pre-warning tree for high-rise buildings

Based on the analysis, a fire prevention risk pre-warning tree for high-rise buildings was established in this study, as shown in Fig. 2.

### Unascertained pre-warning modeling

The unascertained method uses indeterminate mathematics to evaluate systems that exist objectively but are considered to have limited subjective knowledge. The method has been widely used in evaluation research in various fields because of its flexible selection of evaluation factors, evidence-based evaluation process, and accurate and clear evaluation results.

In the evaluation process, subjective omissions or cognition deviations in the evaluator’s understanding may result in the inability to accurately evaluate the objective state or quantitative relationships, thus resulting in uncertainty in the evaluation process. This uncertainty factor is difficult to determine, and human factors are also difficult to quantify, such that a system of this nature can be said to be an uncertain system. In order to quantitatively describe the state of things in an uncertain system and determine the degree of uncertainty, Giordani and Mari (2012) proposed an uncertainty measurement model. This model uses numbers in a specific interval to indicate the degree to which things are in a state of uncertainty. This uncertainty measurement model provides an objective and quantitative method for evaluators, enabling them to deal with uncertain information more accurately and better understand and explain the state of the system during the evaluation process.

**Fig. 3:**
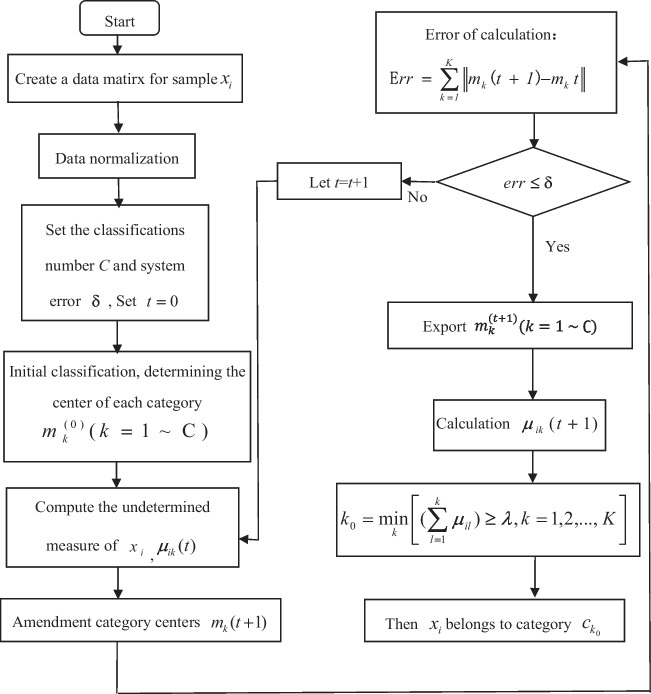
The uncertainty assessment modeling flowchart

The unascertained method is also an effective way to solve engineering uncertainty problems (Zhou et al. 2021). Because of the uncertainty of fire occurrence in high-rise buildings (Cadena et al. 2020), it is feasible to use the unascertained method to model and accurately determine the pre-warning fire prevention risk level of high-rise buildings. Compared with other unascertained methods such as fuzzy model, the method has a higher level of accuracy and calculation intelligence, since the method uses degree for classification rather than 0 or 1 classification, and its assessment results are more realistic. Therefore, it is very suitable for applying to fire risk assessment. The flow chart of unascertained pre-warning modeling is illustrated in Fig. 3 and its scenarios are outlined below (Zhou et al. 2021):

A known sample is ${x}_{i}={\left({x}_{i1,}{x}_{i2},\dots ,{x}_{id}\right)}^{T}$, $i=\text{1,2},\dots n$. *d* is the number of indicators. To divide the sample into *k* categories: ${C}_{1},{C}_{2},\dots ,{C}_{K}$, ${m}_{k}$ is the center vector of class ${C}_{k}\left(k=\text{1,2},\dots ,K\right)$, and ${m}_{k}$ has the same dimension as ${x}_{i}$. ${\mu }_{tk}\left(t\right)$ is the unascertained measure that sample ${x}_{i}$ belongs to the *k*^*th*^ class at iteration *t*.

Let ${m}_{k}^{\left(0\right)}$ be the center vector of the initial classification ${C}_{k}$:

1 $${m}_{k}^{\left(0\right)}={\left({m}_{k1}^{\left(0\right)},{m}_{k2}^{\left(0\right)},\dots ,{m}_{kd}^{\left(0\right)}\right)}^{T}$$

$$\overline{m }=\frac{1}{C}\sum_{k=1}^{C}{m}_{k}$$

2 $$\text{Namely}, \overline{m }=\left({\overline{m} }_{1},{\overline{m} }_{2},\dots ,{\overline{m} }_{d}\right)$$

3 $$\begin{array}{cc}\text{Let }{\sigma }_{j}^{2}=\frac{1}{C}\sum \limits_{k=1}^{C}{\left({m}_{kj}-{\overline{m} }_{j}\right)}^{2},& 1\le j\le d,\end{array}$$

Variance ${\sigma }_{j}^{2}$ reflects the discreteness of class centers ${m}_{1},{m}_{2},\dots ,{m}_{K}$ on feature j.

4 $${w}_{j}={\sigma }_{j}^{2}/\sum_{j=1}^{d}{\sigma }_{j}^{2}$$

${w}_{j}$ satisfies:$0\le {w}_{j}\le 1$, $\sum \limits_{j=1}^{d}{w}_{j}=1$. ${w}_{j}$ is the classification weight of feature *j* for a given classification.

Equation (5) can be used to calculate the possibility measure that sample ${x}_{i}$ belongs to the *k*^*th*^ class:

5 $${\mu }_{ik}=\mu \left({x}_{i}\upepsilon {C}_{k}\right)=\frac{1}{{\rho }_{ik}}/\sum_{k=1}^{K}\frac{1}{{\rho }_{ik}}$$

In Eq. (5), $$0\le {\mu }_{ik}\le 1\cap \sum \limits_{k=1}^{K}{\mu }_{ik}=1$$

6 $${\rho }_{ik}=\sqrt{\sum_{j=1}^{d}{w}_{j}\bullet {\left({x}_{ij}-{m}_{kj}\right)}^{2}}$$

${\rho }_{ik}$ is the distance measure between sample ${x}_{i}$ and the class center of *k*.

The new class center vector ${m}_{k}\left(new\right)$ can be determined by ${\mu }_{ik}$ as follows:

7 $$\begin{array}{cc}m_k\left(new\right)=\sum \limits_{i=1}^n\mu_{ik}\bullet x_i/\sum \limits_{i=1}^n\mu_{ik},&k=\text{1,2},\;.\;.\;.,\;K\end{array}$$

We can replace the initial class center vector ${m}_{k}^{\left(0\right)}$ with the new class center ${m}_{k}\left(new\right)$ and recalculate the unascertained measure ${\mu }_{ik}$ that sample ${x}_{i}$ belongs to the *k*^*th*^ class.

8 $${k}_{0}=\underset{k}{\text{min}}\left[(\sum_{l=1}^{k}{\mu }_{il})\ge \lambda ,k=\text{1,2},\cdots K\right]$$

*λ* is the confidence value.

If ${\mu }_{ik}$ satisfies Eq. (8), then sample ${x}_{i}$ belongs to class ${C}_{k0}$.

Studies have shown that the effect is favorable when 0.5 < *λ* < 1 (Zhou et al. 2021).

## Case study

To verify the feasibility of the fire prevention risk pre-warning model for high-rise buildings established in this study, nine typical high-rise civil buildings were taken as examples for empirical analysis: a modern building (X1), a high-rise building (X2), a high-rise hotel (X3), a university building consisting mainly of classrooms and lecture theaters (X4), an administration building at a university (X5), a residential and commercial building (X6), a residential building (X7), a hospital building (X8), and a public building (X9). The brief information on each building is shown in Table 6.

**Table 6:** Overview of each high-rise building

Building overview	X1^*^	X2^**^	X3^***^	X4	X5	X6	X7	X8	X9
Building type	Comprehensive business building	Residential building (podium commercial)	Hotel	Teaching building	Complex laboratory building	Commercial and residential building	Residential building	Medical nursing home	Office building
building story	− 3F ~ 51F	− 1F ~ 33F	− 1F ~ 18F	− 1F ~ 12F	− 1F ~ 10F	− 2F ~ 24F	− 2F ~ 18F	− 1F ~ 25F	− 1F ~ 9F
Floor area (m^2^)	95,700	189,090	9348	25,990	21,300	84,309	12,000	160,000	11,000

^*^Cao (2008)

^**^Niu (2007)

^***^Cao (2013)

The fire risks of these case buildings were evaluated by five experts according to the actual risk profile of the in-use high-rise buildings in conjunction with the fire code. In this study, criteria were developed for potential participating experts to ensure that the information they provide is valuable. First, participants should have an educational background in the building fire protection profession with a bachelor’s degree or higher, which will ensure the value of the feedback collected. Additionally, the experts need to have been involved in building firefighting-related work for more than 5 years and have earned a senior-level designation, which ensures the accuracy of the data provided from a practical standpoint. To ensure the pre-warning results are more accurate, the initial data were standardized, and the standardized final scores are shown in Table 7.

**Table 7:** Fire prevention risk data of typical high-rise buildings

Samples									
Value indicators	X1	X2	X3	X4	X5	X6	X7	X8	X9
Main factor 1.1	0.9643	0.1786	0.5714	0.9643	1.0000	0.0000	0.0357	0.8571	0.1071
Main factor 1.2	1.0000	0.1707	0.3171	0.9268	0.8537	0.0000	0.1707	0.8049	0.5610
Main factor 2.1	0.6500	0.1000	0.4000	0.6500	1.0000	0.5000	0.6500	0.9000	0.0000
Main factor 2.2	0.7750	0.8000	0.0000	1.0000	1.0000	0.6750	0.9000	0.9750	0.4000
Main factor 3.1	1.0000	0.7727	0.0000	0.3864	0.7955	0.5682	0.5455	0.8636	0.7727
Main factor 3.2	1.0000	0.8000	0.0000	0.3000	0.6667	0.3667	0.3667	0.7667	0.3333
Main factor 3.3	0.6667	0.9444	0.0000	0.5833	1.0000	0.7778	0.6389	0.9722	0.5278
Main factor 4.1	0.6944	0.8611	0.9722	1.0000	0.7222	0.0000	0.7778	0.3611	0.1667
Main factor 5.1	0.8276	1.0000	0.6552	0.3793	0.7241	0.0000	0.3448	0.5172	0.7241
Main factor 6.1	1.0000	0.7667	0.1667	0.0000	0.7667	0.2333	0.1333	0.7000	0.2667
Main factor 6.2	1.0000	0.7667	0.1667	0.0000	0.7333	0.2333	0.1333	0.7333	0.6000

This research applied the unascertained pre-warning model to determine the pre-warning fire prevention risk rating for the nine above-mentioned buildings. The warning results were divided into four categories: very dangerous, dangerous, normal, and safe.

First, the main parameters of the model were established. According to the classification criteria of Eq. (8), combined with literature research (Zhou et al. 2021), it was found that the unascertained pre-warning effect is the best when the confidence degree *λ* is 0.6 ~ 0.7, and the system error $\varepsilon \text{is }0.001$. As such, in this paper, through experiments, it is found that the classification effect is best when λ is taken as 0.62; therefore, *λ* is taken as 0.62 and the classification accuracy is taken as the system default value of 0.001 in this paper.

## Results

By taking the collected data into the pre-warning model, this study calculated the comprehensive pre-warning fire risk profile for 9 high-rise buildings. The unascertained pre-warning system is not only capable of obtaining accurate pre-warning results, but it can also obtain the subordinate degree of each pre-warning object. The distribution of each sample by fire risk rating category is shown in Table 8.

**Table 8:** Distribution of each sample by fire risk rating category

	Sample								
Risk categories	X1	X2	X3	X4	X5	X6	X7	X8	X9
Very dangerous	0.1282	0.3014	0.2937	0.2008	0.0928	0.3107	0.3111	0.0754	0.3091
Dangerous	0.1283	0.3012	0.2938	0.2010	0.0928	0.3104	0.3109	0.0754	0.3090
Normal	0.1292	0.2987	0.2949	0.2028	0.0936	0.3061	0.3069	0.0761	0.3066
Safe	0.6143	0.0987	0.1175	0.3954	0.7208	0.0729	0.0711	0.7732	0.0753

Table 8 shows that X1’s degree of affiliation with the first category, i.e., the degree to which X1 belongs to the first category (very dangerous), is 0.1282 (i.e., the possibility of very dangerous is 12.82%), X1’s degree of affiliation with the second category (dangerous) is 0.1283, its degree of affiliation with the third category (normal) is 0.1292, and its degree of affiliation with the fourth category (safe) is 0.6143. According to the confidence classification criteria expressed in Eq. (8), the comprehensive pre-warning fire risk result for X1 is the fourth category (i.e., safe), given that *λ* = 0.62. Based on Table 8, the final pre-warning results (35 iterations, and a system error *ε* is 0.0008834) are shown in Table 9.

**Table 9:** Sample pre-warning results

Sample	X1	X2	X3	X4	X5	X6	X7	X8	X9
Pre-warning result	Safe	Normal	Normal	Safe	Safe	Dangerous	Dangerous	Safe	Normal

The pre-warning results show that samples X6 and X7 were found to belong to the second category (fire prevention risk rating of dangerous). Samples X2, X3, and X9 were found to belong to the third category (fire prevention risk rating of normal). Samples X1, X4, X5, and X8 were found to belong to the fourth category (fire prevention risk rating of safe).

The pre-warning results for samples X1, X2, and X3 were found to be basically consistent with the results available in the literature (for example, in the literature, the pre-warning fire prevention risk rating for X1 is safe, and that of samples X2 and X3 is relatively safe, i.e., normal) (Cao 2008, 2013; Niu 2007). Meanwhile, the pre-warning results for X4, X5, X6, X7, X8, and X9 were found to be consistent with the actual fire prevention risk condition as assessed by the local fire department. For example, the residential and commercial building, X6, and the high-rise residential building, X7, each received several fire risk rectification notices from the local fire department. The main reason for the high fire risk of X6 is that it is a combined residential/commercial building, and most of the commercial portions of the building are located on the second floor underground, with many fire hazards, a large fire load, inadequate fire partitions, unclear fire evacuation instructions, and so on. Building X7, meanwhile, is a group-rental high-rise residential building, disorderly electrical wiring, untidy fire evacuation passages with obstacles impeding egress, inadequate smoke control and exhaust design, etc., so its fire risk is also high. Although the overall fire risk rating of X2, X3, and X9 is normal, each of these buildings has shortcomings to be rectified with respect to fire prevention design.

For comparative analysis, this study also adopted the K-means clustering method to determine the pre-warning fire prevention risk rating of the nine high-rise civil buildings. K-means clustering is a vector quantization method designed to divide n observations into k clusters. The goal of the method is to organize the data points into clusters such that points within the same cluster are more similar to each other than to points within other clusters. Given a set of n data points $X$={*x*_1_,*x*_2_,…,*x*_n_} in d-dimensional space, the K-means algorithm aims to partition the data into K clusters *S* = {*S*_1_,*S*_2_,…,*S*_k_}. Each cluster *S*_*k*_ is associated with a mean*μ*_*k*_, which is the mean of all the points in cluster. The objective function is:

9 $$\text{arg }\underset{s}{\text{min}}\sum \nolimits_{i=0}^{\text{k}}\sum \nolimits_{x\in {S}_{i}}{\Vert x-{\mu }_{i}\Vert }^{2}$$

*k* is the number of clusters; *x* is a data point; ${S}_{i}$ is the set of points assigned to cluster *i*; ${\mu }_{i}$ is the centroid of cluster *i*; $\Vert x-{\mu }_{i}\Vert$ is the Euclidean distance between a data point *x* and the cluster centroid ${\mu }_{i}$.

As a basic algorithm, K-means clustering has diverse features to quickly partition data and get results (Na et al. 2010). The pre-warning results of the K-means clustering are shown in Table 10.

**Table 10:** Pre-warning results of K-means clustering

Sample	X1	X2	X3	X4	X5	X6	X7	X8	X9
Pre-warning result	Safe	Safe	Dangerous	Very dangerous	Safe	Normal	Normal	Safe	Normal

The pre-warning results of Table 10 show that samples X1, X5, and X8 were found to belong to the fourth category (fire prevention risk rating of safe). Samples X6, X7, and X9 were found to belong to the third category (fire prevention risk rating of normal). Sample X3 was found to belong to the second category (fire prevention risk rating of dangerous). Sample X4, finally, was found to belong to the first category (fire prevention risk rating of very dangerous). As these results show, there is a clear deviation between the K-means clustering method and the unascertained clustering pre-warning results in Table 9. For example, the pre-warning result for sample X3 is “dangerous,” a rating inconsistent with the study in the literature of “normal,” and the pre-warning result for sample X4 is “very dangerous,” a rating that is obviously incorrect. The pre-warning results for samples X2, X6, and X7 were also found to be inconsistent with the actual fire prevention risk condition (based on the results of the inspection by the local fire department).

## Discussion and conclusion

The findings of this study reveal that the unascertained model as a means of assessing building fire risk—with this method featuring a comprehensive evaluation index system with 11 indicators—stands apart from conventional approaches such as the traditional BP neural network and others. Unlike conventional approaches, the proposed evaluation process does not lead to dimensional catastrophes, and the number of indicators employed does not influence the reliability of the evaluation outcomes. The unascertained pre-warning model also appraises the degree of affiliation with a given level or category, assigning the evaluated sample a value between 0 and 1 accordingly. This nuanced approach, in contrast with traditional methods’ binary (0 or 1) approach, aligns more accurately with real-life fire risk scenarios in civil buildings. This distinction, in particular, which underscores the superiority of the unascertained model, sets it apart from its traditional counterparts.

Furthermore, in contrast to the intricate manual calculations involved in the fuzzy clustering evaluation method, the unascertained model streamlines the process by integrating computer technology. This enhancement renders the evaluation process more feasible and user-friendly.

In summary, although the pre-warning of building fire risk, which is inherently uncertain, poses a significant engineering challenge, this study effectively addresses this issue by transforming the building fire risk uncertainty into a relatively specific problem through the unascertained model of the degree of affiliation. This approach provides insights into the likelihood of each fire scenario occurring, thereby converting an otherwise uncertain situation into a relatively certain one. This strategic transformation via the unascertained model constitutes a novel and practical approach to solving building fire risk assessment issues.

Accurate pre-warning of fire prevention risk is the key to preventing or controlling fire events in high-rise buildings. In this study, considering the characteristics of the various types of high-rise civil buildings in use, a fire prevention risk pre-warning framework was built based on the typical event analysis and the unascertained method. The notable research results and conclusions are summarized as follows:

1. Using the event analysis method in consultation with the building fire prevention code, the notion of a “fire prevention risk pre-warning tree for high-rise buildings” was developed that provides a more scientific index system for accurate pre-warning of high-rise building fire prevention risk.
2. To address the inherent uncertainty of fire occurrence in high-rise civil buildings, a pre-warning model was built based on the unascertained method for high-rise building fire risk, where the proportions of indicators were determined based on the data itself rather than on expert opinion, thereby minimizing the impact of human error and subjectivity.
3. Taking nine typical high-rise civil buildings data as examples, case study was conducted, and the feasibility and superiority of the pre-warning model were verified by comparing the warning results with that of the K-means clustering pre-warning method.

The study results demonstrate the reliability of the developed pre-warning model for preventing or controlling fire disasters in high-rise buildings. The pre-warning result can provide a reference for insurers in formulating reasonable fire insurance rates for high-rise buildings. Meanwhile, the “fire prevention risk pre-warning tree for high-rise buildings” can play an important role in high-rise civil building fire disaster prevention and control. Nevertheless, this study needs more international cases to confirm the validity of the findings in different regions or countries, which may impact the applicability of the results. In the future, the focus of fire prevention in high-rise buildings should be more concerned with developing precise and uniform standards to regulate and guide the management of facilities and the design of fire protection systems within high-rise buildings.

This study developed a fire pre-warning framework for high-rise buildings containing 11 key factors, which provided a comprehensive evaluation method for systematically evaluating the fire risk of high-rise buildings. In addition, this study used the unascertained model to validate the predictions of the risk analysis results, and this research method provided an accurate risk assessment value, which improved the prediction accuracy of the fire risk. In the innovation perspective, the research methodology of this study entails an innovative unascertained model, which is an efficient computational model, and its use simplifies the complexity of the fire risk calculation process, making the assessment process more efficient. Finally, the application of unascertained model data materializes the complex and uncertain fire risk, which provides new ideas for the study of uncertainty in complex systems.

Meanwhile, The pre-warning tree for fire risk in high-rise buildings established in this study provides a systematic approach to assessing fire risk factors in high-rise buildings, and this study is of great practical significance and will have a far-reaching impact on practitioners in the industry. From a practical point of view, the early warning tree model proposed in this study will assist engineers and safety personnel in systematically identifying fire hazards in high-rise buildings, prioritizing the occurrence of fires, and developing targeted fire prevention and control measures. In addition, the pre-warning tree model developed in this study will guide designers to optimize the building structure and layout, such as the layout of firefighting ducts, during the design phase of high-rise buildings.

Therefore, the findings of this study are instructive to practitioners in the industry, who should be encouraged to understand and master the warning tree model and how to properly use the unascertained method to determine the risk level. Practitioners can learn about and acquire these skills by attending seminars and professional skills training courses.

## Data Availability

The author confirms that the primary data generated or analyzed during this study are included in this article. Additional data are also available from the authors upon reasonable request.

## Appendix

11

**Table 11:** Fire prevention grade and decorative materials smokiness scoring of high-rise buildings

Fire resistance of decorative materials	Actual risk condition	Value range
Class A	Safe	80 ~ 100
Classes A and B1	Normal	60 ~ 79
Class B1	Dangerous	45 ~ 59
Class B2 or class B3	Very dangerous	0 ~ 44

12

**Table 12:** Reliability of electrical equipment scoring of high-rise buildings

Building electrical equipment condition	Actual risk condition	Value range
Equipment of well-known brands, reliable performance, regular inspection	Safe	80 ~ 100
Equipment is of generic brand, average performance, regular inspection	Normal	60 ~ 79
Equipment of unknown brand, poor performance, no regular inspection	Dangerous	45 ~ 59
Frequent breakdowns of equipment and lack of regular inspections	Very dangerous	0 ~ 44

13

**Table 13:** Safety facilities of power distribution equipment scoring of high-rise buildings

Power distribution equipment condition	Actual risk condition	Value range
The capacity of the equipment fully meets the requirements of electricity, the installation location is dry and ventilated, and there are measures to prevent dust, sunshine, short-circuit, lightning, and so on	Safe	80 ~ 100
The capacity of the equipment meets the requirements of electricity, the installation location is dry, and there are measures to prevent short-circuit and lightning	Normal	60 ~ 79
The capacity of the equipment barely meets the requirements of electricity, there are some measures to prevent short-circuit and lightning	Dangerous	45 ~ 59
The capacity of the equipment cannot meet the requirements of electricity, no short-circuit protection, lightning protection, and other measures	Very dangerous	0 ~ 44

14

**Table 14:** Fire resistance of wires/cables scoring of high-rise buildings

Fire resistance of wires/cables	Actual risk condition	Value range
Class A	Safe	80 ~ 100
Classes A and B1	Normal	60 ~ 79
Class B1	Dangerous	45 ~ 59
Class B2 or class B3	Very dangerous	0 ~ 44

15

**Table 15:** The fire load density scoring of high-rise buildings

The fire load density condition	Actual risk condition	Value range
Very low fire load density (*f* < 20kg/m^2^)	Safe	80 ~ 100
low fire load density (20 ≤ *f* < 35kg/m^2^)	Normal	60 ~ 79
High fire load density (35 ≤ *f* < 50kg/m^2^)	Dangerous	45 ~ 59
Very high fire load density (*f* > 50kg/m^2^)	Very dangerous	0 ~ 44

*f* is the fire load density

16

**Table 16:** Horizontal and vertical fire division scoring of high-rise buildings

Horizontal and vertical fire division condition	Actual risk condition	Value range
The horizontal and vertical fire division fully meet code requirements	Safe	80 ~ 100
The horizontal and vertical fire division basically meet code requirements	Normal	60 ~ 79
Individual floor horizontal fire division do not meet code requirements	Dangerous	45 ~ 59
The horizontal and vertical fire division do not meet code requirements	Very dangerous	0 ~ 44

17

**Table 17:** The intact condition of smoke prevention and exhaust facilities scoring of high-rise buildings

Smoke prevention and exhaust facilities condition	Actual risk condition	Value range
Smoke evacuation facilities are complete, in good working order and regular inspection	Safe	80 ~ 100
There are some smoke evacuation facilities, in working order and regular inspection	Normal	60 ~ 79
Only a few smoke evacuation facilities, no regular inspection	Dangerous	45 ~ 59
No smoke evacuation facilities	Very dangerous	0 ~ 44

18

**Table 18:** The design rationality of smoke prevention and exhaust system scoring of high-rise buildings

Smoke prevention and exhaust system condition	Actual risk condition	Value range
Smoke height ≥ 2.2 m, smoke exhaust air velocity < 10m/s, residual pressure value ≥ 50Pa, the critical smoke exhaust volume of the smoke vent meets the requirements	Safe	80 ~ 100
Smoke height ≥ 2.1 m, smoke exhaust air velocity < 10m/s, residual pressure value ≥ 40Pa	Normal	60 ~ 79
Smoke height < 2.1 m, smoke exhaust air velocity ≥ 10m/s, residual pressure value ≥ 25Pa	Dangerous	45 ~ 59
Smoke height < 1.2 m, smoke exhaust air velocity > 10m/s, residual pressure value < 25Pa	Very dangerous	0 ~ 44
